# Elevated Rate of Fixation of Endogenous Retroviral Elements in Haplorhini *TRIM5* and *TRIM22* Genomic Sequences: Impact on Transcriptional Regulation

**DOI:** 10.1371/journal.pone.0058532

**Published:** 2013-03-14

**Authors:** William E. Diehl, Welkin E. Johnson, Eric Hunter

**Affiliations:** 1 Emory Vaccine Center, Yerkes National Primate Research Center, Emory University, Atlanta, Georgia, United States of America; 2 Department of Pathology, Emory University, Atlanta, Georgia, United States of America; 3 Biology Department, Boston College, Chestnut Hill, Massachusetts, United States of America; INRA, France

## Abstract

All genes in the *TRIM6*/*TRIM34*/*TRIM5*/*TRIM22* locus are type I interferon inducible, with *TRIM5* and *TRIM22* possessing antiviral properties. Evolutionary studies involving the *TRIM6*/*34*/*5*/*22* locus have predominantly focused on the coding sequence of the genes, finding that *TRIM5* and *TRIM22* have undergone high rates of both non-synonymous nucleotide replacements and in-frame insertions and deletions. We sought to understand if divergent evolutionary pressures on *TRIM6/34/5/22* coding regions have selected for modifications in the non-coding regions of these genes and explore whether such non-coding changes may influence the biological function of these genes. The transcribed genomic regions, including the introns, of *TRIM6*, *TRIM34*, *TRIM5*, and *TRIM22* from ten Haplorhini primates and one prosimian species were analyzed for transposable element content. In Haplorhini species, *TRIM5* displayed an exaggerated interspecies variability, predominantly resulting from changes in the composition of transposable elements in the large first and fourth introns. Multiple lineage-specific endogenous retroviral long terminal repeats (LTRs) were identified in the first intron of *TRIM5* and *TRIM22*. In the prosimian genome, we identified a duplication of *TRIM5* with a concomitant loss of *TRIM22.* The transposable element content of the prosimian *TRIM5* genes appears to largely represent the shared Haplorhini/prosimian ancestral state for this gene. Furthermore, we demonstrated that one such differentially fixed LTR provides for species-specific transcriptional regulation of *TRIM22* in response to p53 activation. Our results identify a previously unrecognized source of species-specific variation in the antiviral TRIM genes, which can lead to alterations in their transcriptional regulation. These observations suggest that there has existed long-term pressure for exaptation of retroviral LTRs in the non-coding regions of these genes. This likely resulted from serial viral challenges and provided a mechanism for rapid alteration of transcriptional regulation. To our knowledge, this represents the first report of persistent evolutionary pressure for the capture of retroviral LTR insertions.

## Background

The human genome encodes in excess of 70 tripartite motif-containing (TRIM) genes. This family of genes is characterized by the presence of a RING domain, one or two B-box domains, and a coiled-coil domain. The C-termini of these genes can consist of one or more of a select number of protein domains, with the majority of the TRIM genes in the human genome containing either a SPRY or PRY-SPRY (B30.2) domain at their C-terminus [1]. SPRY-containing TRIM genes have undergone rapid and extensive amplification via gene duplication and many of these genes display evidence of a history of positive selection [2], [3], [4], [5], [6], [7], [8]. Furthermore, a number of B30.2 domain containing TRIM genes have been implicated in host defense mechanisms owing to their transcriptional induction following interferon or viral stimulation [7], [9], [10] or to their inherent ability to inhibit viral replication [11], [12], [13]. Primate *TRIM6*, *TRIM34*, *TRIM22* and *TRIM5* are all interferon inducible [9], while *TRIM5* and *TRIM22* possess antiviral properties [11], [12], [13].

Studies involving *TRIM5* have revealed antiretroviral activities for the alpha splice variant of this gene (*TRIM5*) from members of all branches of Haplorhini primates, which include hominoid, Cercopithecidae (Old World monkey), and Platyrrhini (New World monkey) species [5], [14], [15], [16], [17], [18], [19], [20], [21], [22], [23]. A more recent report has demonstrated that TRIM5α from Strepsirrhini (prosimian) lineages also possess antiviral function [24]. Antiretroviral activity appears to be a general feature of TRIM5 as cow and rabbit orthologs are capable of blocking retroviral infection [25], [26], [27]. However, antiviral activity of any specific TRIM5/virus combination is governed by features in both TRIM5 and virus, being largely determined by the B30.2 domain within TRIM5 and conformation-dependent motifs presented on the mature viral capsid (CA) cores [20,28,29,30,31,32,33,34,35,36,37,38,39, reviewed in 40].

Several reports have demonstrated an anti-HIV-1 activity for the closely related gene *TRIM22*. The mechanism by which this anti-retroviral activity is exerted has yet to be fully elucidated, but appears to involve suppression of LTR transcription [13], [41], [42]. In addition to the effects on HIV-1 replication, TRIM22 has been shown to inhibit hepatitis B virus and encephalomyocarditis virus replication in tissue culture systems [43], [44].

*TRIM5* and *TRIM22* along with *TRIM6* and *TRIM34* form a locus of four closely related TRIM genes within an olfactory receptor-rich region on chromosome 11 of the human genome. Genetic analysis of this locus indicates that these four genes have evolved by gene duplication from a common ancestral gene [1]. Host genes in direct evolutionary competition with pathogenic invaders are often subject to high levels of positive selective pressures and this is the case for both *TRIM5* and *TRIM22*. Both genes have faced positive selective forces during primate evolution [4], [5], [45], but evolutionary constraints appear to have resulted in a dichotomous situation where along any given primate lineage only one of these genes (either *TRIM5* or *TRIM22*) shows signs of positive selection [4]. Additionally, positive selective pressure on these genes has not been uniformly applied across the gene. The B30.2 region, consistent with its role in mediating viral recognition, has faced positive selective forces, while the tripartite motif-encoding regions have been under purifying selection to maintain function of the RING, B-box and coiled-coil domains [5], [12], [45]. In contrast, over the same period of time *TRIM6* and *TRIM34* have been under constant purifying selection.

**Figure 1:**
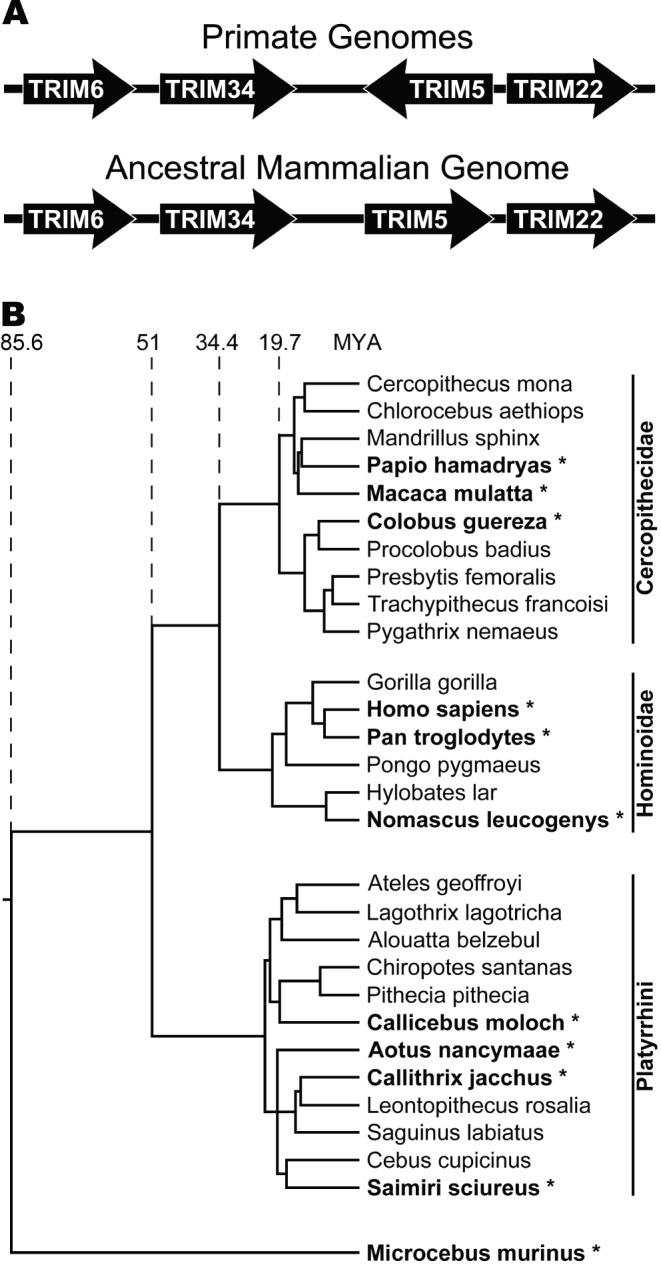
Evolutionary history of *TRIM5* and the primate species involved in this study. (A) Graphical depiction of the *TRIM6/34/5/22* genomic locus of primates as well as a depiction of the hypothetical ancestral mammalian genomic locus. (B) Phylogenetic tree showing the evolutionary relationship of primate species representative of the most prominent genera of primate evolution. The following species were examined in this study: human (*Homo sapiens*), chimpanzee (*Pan troglodytes*), white-cheeked gibbon (*Nomascus leucogenys*), olive baboon (*Papio anubis*), rhesus macaque (*Macaca mulatta*), guereza colobus (*Colobus guereza*), Peruvian red-necked owl monkey (*Aotus nancymaae*), common marmoset (*Callithrix jacchus*), Bolivian squirrel monkey (*Saimiri boliviensis boliviensis*), dusky titi (*Callicebus moloch*), and grey mouse lemur (*Microcebus murinus*). These species are highlighted using ‘*’ as well as bold lettering. This phylogenetic tree was adapted from Bininda-Emonds et al. 2007, and uses the revised dates published with the corrigendum on the original article.

In addition to coding sequence changes, the structure and composition of the *TRIM6/34/5/22* genomic locus has undergone lineage-specific changes during mammalian evolution. The genomic locus of all studied Haplorhini primates has the following gene order: *TRIM6*-*TRIM34*-*TRIM5*-*TRIM22*, where *TRIM5* is present in opposite orientation to the rest of the genes (Figure 1A). This genomic structure differs from that of the ancestral locus, which is maintained in the dog and other species [Diehl WE, unpublished data and 4], where *TRIM5* is found in the same orientation as the other three genes. Rodent genomes have *TRIM5* in the same orientation as primates, thus the inversion event most likely occurred prior to the rodent-primate divergence [6]. In contrast to what is found in primate genomes, *TRIM5* has undergone gene duplication events in rodents that were accompanied by a resultant loss of the *TRIM22* gene. Furthermore, in mice, but not rats, one duplication event resulted in two copies of *TRIM34* being present in the genome [6]. In the cow genome, similar to in rodents, *TRIM5* has undergone a number of gene duplication events in conjunction with a loss of the *TRIM22* gene resulting in five *TRIM5* orthologs as well as three pseudogenes. In contrast to the rodent genomes, the *TRIM5* genes in cows are all present in the positive orientation [4].

A subtler means of genome modification involves insertion and removal of short nucleotide sequences, a phenomenon that often involves transposable elements. In the human genome, the most complete and fully characterized mammalian genome, approximately 43% of the DNA content is derived from retrotransposons, in the form of long interspersed nuclear elements (LINEs), short interspersed nuclear elements (SINEs) and endogenous retroviral (ERV) elements, while DNA transposons account for an additional 2% of the genome [46]. In certain contexts these elements have been shown to potentiate altered transcriptional regulation. LINE elements can modulate normal splicing [47], [48], alter polyadenylation [49], interfere with transcriptional elongation [50], and provide novel promoter activity for neighboring genes [51]. SINE family members, including *Alu* elements, have been shown to have effects upon polyadenylation [52], [53], [54], alternate splicing patterns [54], and translation efficiency [reviewed in 55]. ERV sequences are generally found in the genome either as the mobilization-competent form of two LTRs flanking internal retroviral-derived sequences or solo LTRs that are the product of homologous recombination between the two LTRs. The overwhelming majority of ERV sequences in the human genome are solo LTRs, which contain most of the viral transcriptional regulatory elements [46], [56]. It has been shown that these LTRs can provide cellular genes with primary or alternate promoters, enhancer elements, protein-binding sites, and polyadenylation signals [57], [58], [59], [60].

In order to determine what effects complex evolutionary pressures in the *TRIM6/34/5/22* locus have had on the non-coding genic sequences, we retrieved primate sequences corresponding to the transcribed region of each of these TRIM genes, including introns, from publicly available sequence databases. These were assembled, aligned, and compared among 10 Haplorhini primates (three Hominoid, three Cercopithecidae, and four Platyrrhini) and one prosimian species. In Haplorhini primates, we observed a hierarchy of overall sequence conservation in these genes (*TRIM6 *> *TRIM34 *> *TRIM22 *> *TRIM5*) resulting from sequence insertions and deletions (indels) involving transposable elements. Differences in transposable element content are largely localized to the large first and fourth introns, with the first intron of *TRIM5* and *TRIM22* containing multiple lineage-specific ERV LTRs and dramatic gains and losses of LINE element content in the fourth intron. In contrast, the *TRIM6/34/5/22* locus in prosimians has undergone duplication of *TRIM5* and loss of *TRIM22*, with no accumulation of novel LTR insertions and limited LINE L1 changes in the fourth intron. We further tested the ability of one specific ERV LTR element to alter regulation of the gene it resides in. This element, LTR10D, is present in the first intron of *TRIM22* in Hominoids and Cercopithecidae, but is absent in Platyrrhini. Using quantitative reverse transcriptase polymerase chain reaction (RT-PCR) on RNA from peripheral blood mononuclear cells (PBMCs), we found that *TRIM22* was upregulated in response to p53 stimulation only in the LTR10D containing Old World primates. Based on these findings, we hypothesize that the antiviral TRIM genes of Haplorhini primates have evolved species-specific differences in transcriptional regulation, which are mediated by transposable element sequences in their non-coding regions.

## Results

### *TRIM5* Displays Increased Genetic Diversity Compared to Related TRIM Genes, as a Result of Turnover in Transposable Element Content in Intronic Sequences

To examine evolution of the primate *TRIM6*/*34*/*5*/*22* locus at the genomic level, orthologous sequences were retrieved from NCBI nucleotide sequence databases (see Methods). In total, sequence information from ten Haplorhini and one prosimian species was retrieved from the online databases, including: human (*Homo sapiens*), chimpanzee (*Pan troglodytes*), white-cheeked gibbon (*Nomascus leucogenys*), olive baboon (*Papio anubis*), rhesus macaque (*Macaca mulatta*), guereza colobus (*Colobus guereza*), Peruvian red-necked owl monkey (*Aotus nancymaae*), common marmoset (*Callithrix jacchus*), Bolivian squirrel monkey (*Saimiri boliviensis boliviensis*), dusky titi (*Callicebus moloch*), and grey mouse lemur (*Microcebus murinus*). Thus, the species included in this study represent a broad sampling of Haplorhini lineages as well as a representative prosimian species to serve as an outgroup (Figure 1B). Following identification and compilation of the genomic sequence information for these TRIM genes, multiple sequence alignments were generated for each TRIM gene’s transcribed region (Files S1–S4). Due to differences in sequence composition and genomic architecture of the locus (see below) prosimian sequences were not included in the initial comparisons.

**Figure 2:**
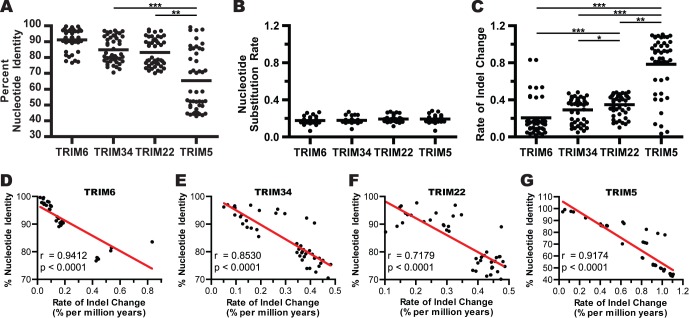
TRIM genes are variably conserved as a result of differential rates of indel turnover. Transcribed genomic sequences of the *TRIM5*, *TRIM6*, *TRIM22*, and *TRIM34* genes were hand aligned. Using the formulas presented in the Materials and Methods, the nucleotide alignments were used to calculate the following statistics for all pairs of nucleotide sequences: the percent nucleotide identity (A), the nucleotide substitution rate (B), and the rate of indel change (C). The rate of change depicted in these figures is calculated as percent per million years, the black circles indicate separate pairwise sequence comparisons and the bars represent mean values. In panels A-C, statistical significance was calculated using the Friedman test, a one-way repeated measures ANOVA without assuming Gaussian distributions and using the Dunn’s post-test to compare all genes against one another. A p-value of less than 0.05 is denoted by *, a p-value less than 0.01 is denoted by **, a p-value less than 0.001 is denoted by ***. For each gene, the correlation between nucleotide identity and the rate of indel change was examined using Spearman’s rank correlation (D-G). The Spearman r and p-values resulting from this analysis are indicated in each panel.

Next, percent nucleotide identity between species was calculated for each TRIM gene. It was observed that the degree of conservation for genomic sequences of genes in the *TRIM6/34/5/22* locus have an inverse correlation with the previously reported dN/dS rate ratios for these genes (Figure 2A) [4], [61]. Thus, conservation of the genomic sequences for the genes in this locus is *TRIM6 *> *TRIM34 *> *TRIM22 *> *TRIM5*. With some species sharing as little as 44% genomic sequence identity. This simple analysis demonstrated that *TRIM5* sequences are significantly less conserved, at the genomic level, than the other genes in this locus. As might be expected, due to the relative size difference and relaxed evolutionary constraints, most of the sequence divergence was isolated in the intronic regions (data not shown).

In order to determine the relative contribution that nucleotide substitutions versus indels have played in creating the divergent levels of sequence conservation between the TRIM genes, the different types of sequence changes were independently analyzed. It was observed that over the course of Haplorhini evolution the genomic sequences for all four TRIM genes have undergone similar nucleotide substitution rates (Figure 2B). In contrast, very different rates of diversification resulting from indels were observed among the individual genes of the *TRIM6/34/22/5* locus (Figure 2C). The average rate of change due to indels in *TRIM5* is significantly greater compared to all of the other genes, while the rate in *TRIM22* was found to be significantly greater than either *TRIM6* or *TRIM34*. While it is informative to compare the average rate of indel change in these genes, it should also be highlighted that the calculated rate between any two species can vary widely (Figure 2C). This is especially true for *TRIM5*, where there is a large variance in the observed rate of indel-associated change. When the pairwise comparisons are separated by the evolutionary relationship of the species involved (Figure S1A-D) it becomes apparent that the rate of indel change in these genes is lineage specific. For example, in contrast to what is observed in other lineages, Old World primate *TRIM5*s display remarkably low rates of indel change, comparable to what is observed for the other TRIM genes. In spite of these lineage-specific differences, a strong inverse correlation between the rate of change due to indels and the percent nucleotide identity was observed for all TRIM genes in this analysis (Figure 2D–G). In contrast, no correlation between the nucleotide substitution rate and the percent nucleotide identity was found (Figure S2A-D). These observations strongly suggest that indels have been the main generator of sequence diversity in these genes during primate evolution.

**Figure 3:**
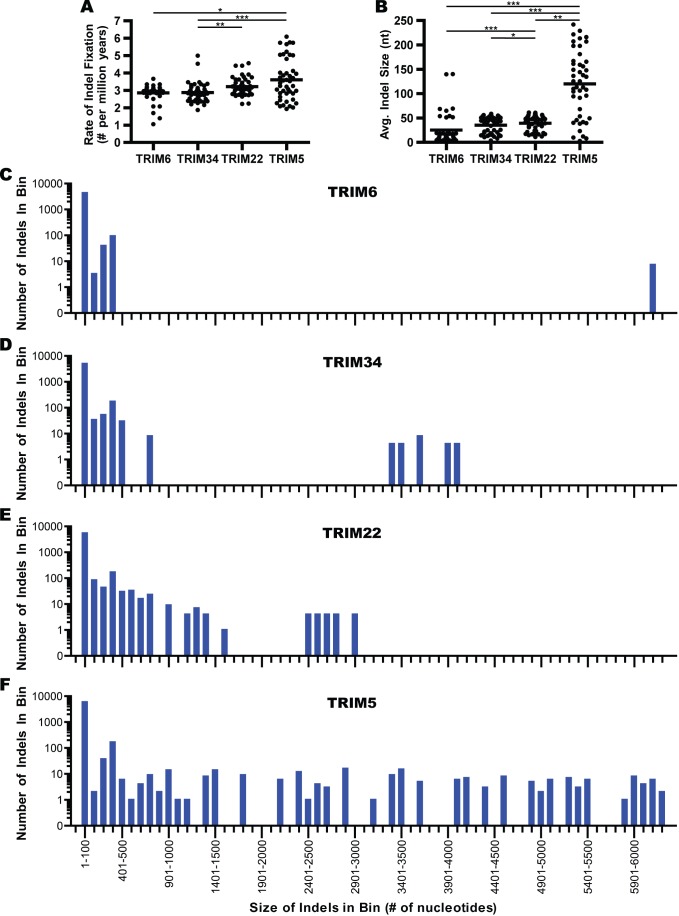
*TRIM5* and *TRIM22* display elevated rates of indel fixation as well as fixation of larger indels. Using the formulas presented in the Materials and Methods, the nucleotide alignments were used to calculate the following statistics for all pairs of nucleotide sequences: the rate of indel creation (A) and the average indel size (B). Dots indicate separate pairwise sequence comparisons and the black bars represent mean values. Statistical significance was calculated using the Friedman test, a one-way repeated measures ANOVA without assuming Gaussian distributions and using the Dunn’s post-test to compare all genes against one another. A p-value of less than 0.05 is denoted by *, a p-value less than 0.01 is denoted by **, a p-value less than 0.001 is denoted by ***. Indels present in pairwise comparisons of each gene were separated by size and placed into a corresponding 100 nucleotide ‘bin’. The number of indels present in each bin is depicted for *TRIM6* (C), *TRIM34* (D), *TRIM22* (E), and *TRIM5* (F).

The increased rates of sequence divergence resulting from indels observed for *TRIM5* and *TRIM22* could be explained by a proportionately greater accumulation of novel indels, the presence of one or more disproportionately large indels, or a combination of both. To assess the contribution of accumulation of novel indels, the rate of indel fixation was estimated in pairwise fashion by dividing the number of indels present between the sequences of two species by the estimated time since the last common ancestor (Figure 3A). This analysis revealed a subtle but statistically significant elevation in the rate of indel accumulation for *TRIM5* and *TRIM22* compared to *TRIM6* or *TRIM34*. To examine the contribution of indel size to the differential rate of nucleotide divergence, the average and median indel sizes for each gene were calculated (Figures 3B and data not shown). Widely divergent average indel sizes were found for these genes, with the average indel size in *TRIM5* genes being significantly larger and the average indel size in *TRIM6* significantly smaller than the other genes. In contrast, all TRIM genes examined were found to have a median indel size of approximately 2 nucleotides.

The observed difference between median and average indel size suggests an influence of proportionally few large indels in driving the sequence divergence rates of these genes. For a more detailed analysis of this, indels from each gene were separated into 100 nucleotide bins of increasing indel size. The number of binned indels for each gene are depicted on a log_10_ scale in Figure 3C–F. This analysis highlights the fact that the overwhelming majority of indels are between 1 and 100 nucleotides in size for all TRIM genes examined, with between 81% and 87% of indels being under 10 nucleotides in size. Where these genes differ is in the number and size of indels greater than 300 nucleotides. *TRIM6* has very few large indels (>300 nucleotides), *TRIM22* and *TRIM34* have intermediate numbers, while *TRIM5* has, by far, the greatest number of large indels. From this data, we conclude that the disparate nucleotide conservation rates observed in these genes is the result of a combination of gene-specific variation in rates of indel accumulation and the differential fixation of large indels.

To ask whether the presence of variable numbers of large indels reflected accumulation of transposable elements, RepeatMasker software was used to identify transposable elements present in each of the TRIM genes. This analysis revealed that these genes contain members of all the major families of primate transposable elements, including LINEs, SINEs (MIRs and *Alu* elements), as well as DNA transposons and endogenous retroviral elements. Examples of all classes of LINEs, SINEs, and DNA transposons identified in this analysis are present in the genomes of both Haplorhini and Strepsirrhini primates, even if the specific insertions characterized here are not. In contrast, several of the endogenous retroviral LTRs identified in this locus are restricted to specific primate lineages.

**Figure 4:**
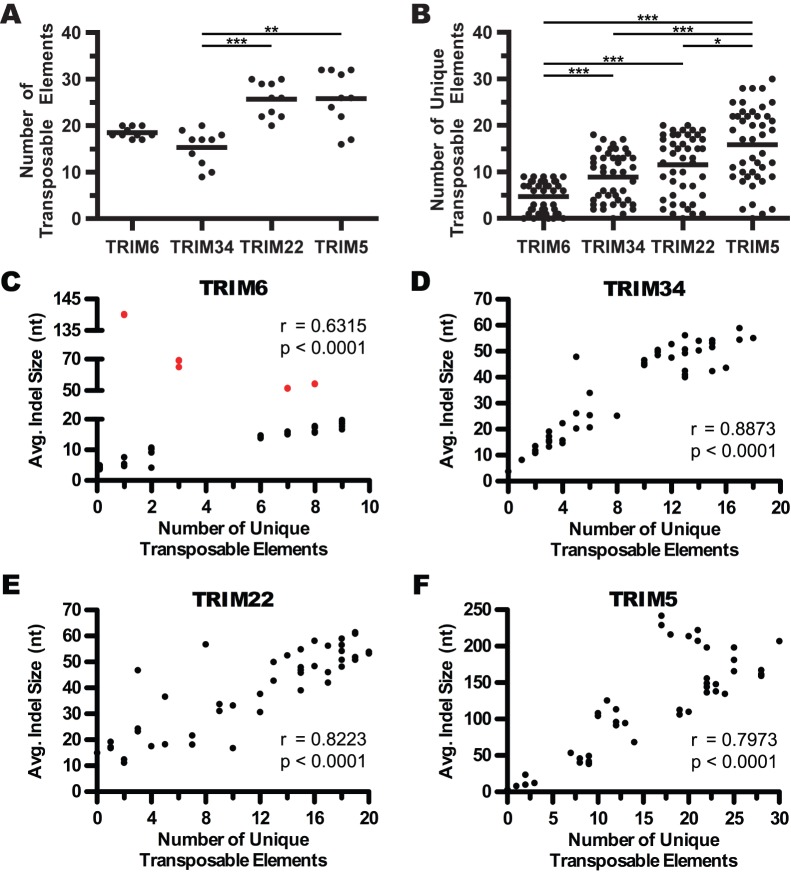
*TRIM5* and *TRIM22* contain more transposable elements than *TRIM6* or *TRIM34*. The absolute number of transposable elements present in each TRIM gene was tallied for each species and the results are depicted in panel (A). Black dots represent the number of transposable elements found in a given primate species and the black bar represents the mean value. The quantitation shown in (A) was performed without regard for identity or conservation of the elements present, therefore the number of novel transposable elements was considered. In panel (B), the number of unique transposable elements present in pairwise comparisons of each TRIM gene is shown. The black dots indicate number of elements in an individual pairwise sequence comparison and the black bar represents the mean value. In panels (A) and (B), statistical significance was calculated using the Friedman test, a one-way repeated measures ANOVA without assuming Gaussian distributions and using the Dunn’s post-test to compare all genes against one another. A p-value of less than 0.05 is denoted by *, a p-value less than 0.01 is denoted by **, a p-value less than 0.001 is denoted by ***. Correlations between average indel size and the number of unique transposable elements were examined *TRIM6* (C), *TRIM34* (D), *TRIM22* (E), and *TRIM5* (F). Statistical significance was assessed using Spearman’s rank correlation the r and p-values resulting from this analysis are indicated in each panel. Comparisons involving colobus *TRIM6*, which contains a 6-kb LINE L1 element insertion, are indicated with red dots.

Without regard to classification, the number of transposable elements identified in the different TRIM genes from each primate species was totaled (Figure 4A). It was found that *TRIM22* and *TRIM5* have significantly more transposable elements than *TRIM6* and *TRIM34*. However, this analysis ignores the nature or conservation of the transposable elements, and therefore the number of unique elements found in pairwise comparisons of sequences was also quantified (Figure 4B). This analysis revealed that, in primates, *TRIM6* has the fewest lineage-restricted transposable element insertions, *TRIM34* and *TRIM22* have an intermediate number, and *TRIM5* has significantly more of these non-conserved transposable elements than all of the other genes. Furthermore, when these data were corrected for the evolutionary distances between these comparisons, similar results were observed with the same trend toward increasing rates of transposable element turnover going from *TRIM6* to *TRIM5* (data not shown). Comparing the number of unique transposable elements in each of the TRIM genes with the average indel size yields strong positive correlations (Figure 4C–F). This correlation for *TRIM6* was found to be significant in spite of the fact that colobus *TRIM6* contains a novel LINE L1 element of approximately 6 kilobases in size, resulting in abnormally large average indel sizes in comparisons involving this species, as indicated with red dots (Figure 4C). Taken together, these analyses point to a striking involvement of transposable elements in the evolution of genes in the *TRIM6/34/5/22* locus, especially those genes whose coding sequences were under positive selective pressures in Haplorhini evolution.

### Lineage-specific Transposable Element Differences Localize to the First and Fourth Introns and the Difference in TE Composition in these Regions is Dominated by ERV LTR and LINE L1, Respectively

**Figure 5:**
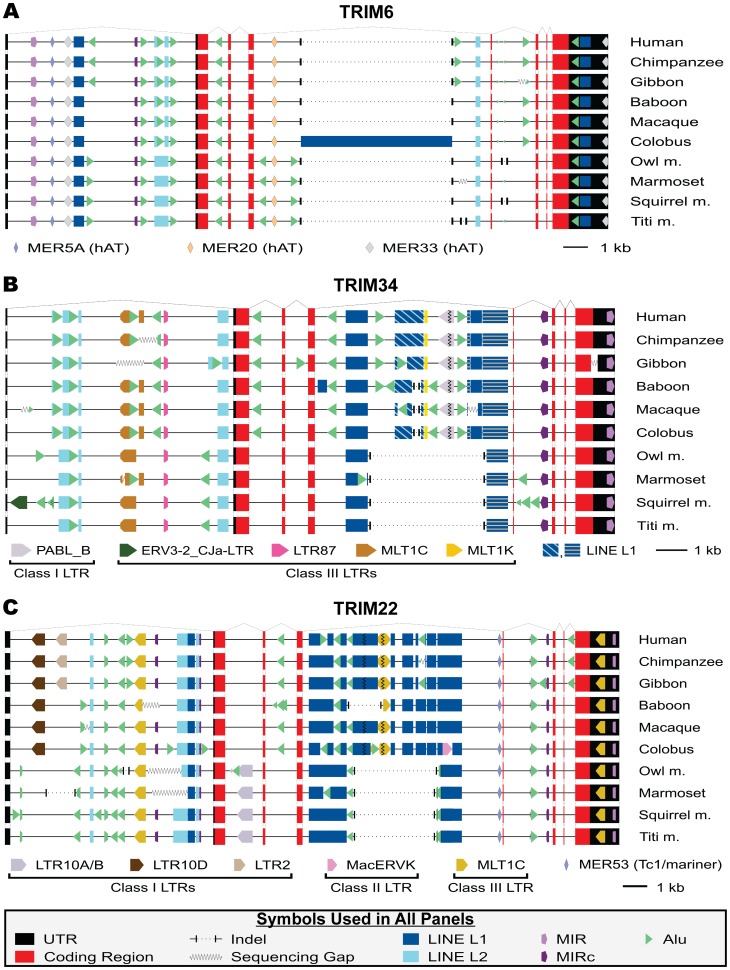
Graphical depiction of the genomic structure and location of transposable elements in the TRIM6, TRIM34, and TRIM22 genes. RepeatMasker was used to identify repetitive elements present in the genomic TRIM gene sequences and these elements were mapped onto the multiple sequence alignments. Graphical representations of the exon/intron structure as well as the various transposable elements found in *TRIM6* (A), *TRIM34* (B), or *TRIM22* (C) are shown. Figures are drawn to approximate scale, with a 1 kb scale bar shown in the legend of each panel. Symbols common to all genes analyzed are shown at the bottom of the figure, while symbols representing non-conserved transposable elements are shown in the panel in which they are present.

**Figure 6:**
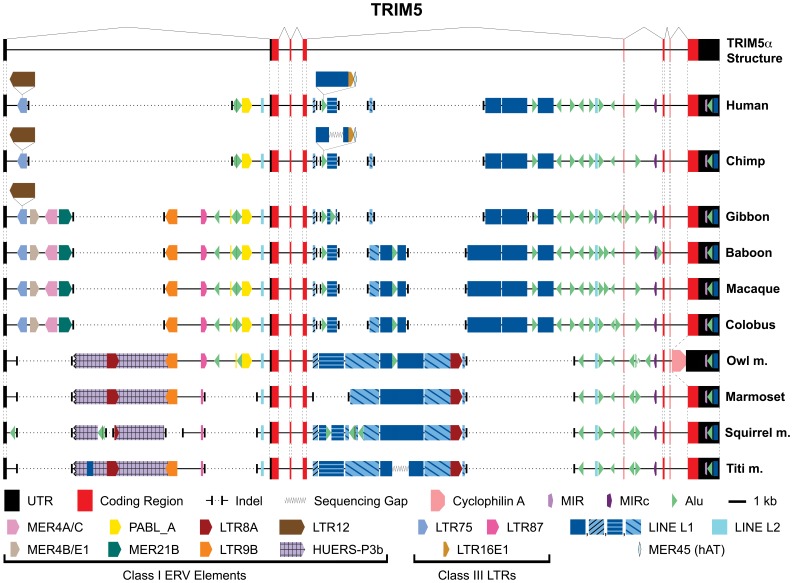
Graphical depiction of the genomic structure and location of transposable elements in the *TRIM5*. In the same fashion as Figure 5, repetitive elements were identified in *TRIM5* genes using RepeatMasker and were mapped onto the exon/intron structure of *TRIM5*. The figure is drawn to approximate scale, with a 1 kb scale bar in the legend. The top most structure represents the exon/intron structure of the gene and all subsequent structures superimpose the unique transposable elements and/or deletions specific to the indicated primate species. Symbols representing all transposable elements are at the bottom of the figure.

Transposable elements identified by RepeatMasker were then mapped back onto the multiple sequence alignments. The position and orientation of these transposable elements in relation to the exon/intron structure of the four TRIM genes are depicted in Figures 5 and 6. This analysis makes starkly clear the difference in magnitude of the genetic variability between the genes of the *TRIM6/34/5/22* locus and highlights both the spatial location and involvement of specific transposable element families resulting in this variability.

#### *TRIM6*

In the species sampled, there are few differences in transposable element content between Haplorhini *TRIM6* sequences (Figure 5A). *Alu* elements account for the majority of lineage-specific transposable elements in *TRIM6* where nine of these elements are found in a lineage-restricted manner. All nine of these *Alu* elements are present in multiple related species. Thus, all of these *Alu* insertions must have occurred prior to speciation and therefore can be inferred to be fixed in the species from which they are identified. We will refer to such elements shared by multiple species as being fixed throughout. In addition to the multiple lineage-specific *Alu* insertions, two other changes involving transposable elements were detected in *TRIM6* sequences. In the fifth intron of Owl and Squirrel monkeys a deletion resulted in the loss of *Alu*-derived sequence. Also, as previously mentioned, the fourth intron of colobus contains a novel and nearly intact LINE L1 element. The colobus LINE L1 insertion is representative of many events in the *TRIM6/34/5/22* locus in which a lineage-specific change was identified in a single species. Except for humans, each species analyzed is represented by the genome of a single individual, thus no inference can be made as to the prevalence of the observed insertions or deletions in the population. Such events could be fixed, present only in the individual sampled, or present at some frequency in between these extremes.

#### *TRIM34*

In Haplorhini primates, *TRIM34* has more lineage-restricted transposable element content than *TRIM6* (Figure 5B). For instance, there are 25 independent *Alu* insertions in *TRIM34*, with only one fixed in all Haplorhini species sampled. The other 24 are lineage-restricted with 8 fixed in multiple species and 16 found only in a single species. Additionally, two deletion events were identified in the fourth intron of Haplorhini *TRIM34*. One removes approximately 3,400 nucleotides of Platyrrhini intronic sequence, while the other removes approximately 225 nucleotides from Old World (baboon, macaque and colobus) monkeys. In contrast to the *TRIM6* sequences, which are devoid of ERV content, *TRIM34* contains five ERV LTR elements. Three of these are found to be lineage-restricted, with one (ERV3-2_CJa-LTR) being a novel insertion found in the squirrel monkey genome. The other two ERV elements (PABL_B and MLT1K) appear to have been lost from Platyrrhini as a consequence of the large deletion event in the fourth intron. Finally, one novel LINE L1 insertion was identified in the fourth intron of baboon *TRIM34*.

#### *TRIM22*

The number of lineage-restricted transposable elements and complexity of intronic diversity of Haplorhini *TRIM22* sequences is similar to that of *TRIM34* (Figure 5C). *TRIM22* sequences contain 27 independent *Alu* insertions. Four of these are conserved in all species examined, while 23 *Alu* insertions are found to be lineage-restricted; 12 of these fixed in multiple species and 11 found only in a single species. Intron four of Platyrrhini *TRIM22* contains a deletion of approximately 3,200 nucleotides, similar in size to the deletion in the fourth intron of Platyrrhini *TRIM34*. Three additional large deletions occurred in the first and fourth intron of *TRIM22*, where each is restricted to a single species. Similar to *TRIM34*, there are five lineage-restricted ERV LTR elements found in *TRIM22*, with one (MacERVK) being recently inserted into a single species, colobus in this instance, and one LTR (MLT1C) lost from Platyrrhini as a result of the large deletion in intron four. In contrast to *TRIM34*, three ERV LTRs inserted and became fixed during Haplorhini evolution. An LTR10D element became fixed in the first intron of *TRIM22* early in Old World primate evolution and is present in all extant Cercopithecidae sampled. Later, an LTR2 element inserted and became fixed in the ancestral hominoid lineage and is found immediately downstream of the LTR10D element in all hominoid species sampled. Finally, an LTR element that appears to be a recombinant between LTR10A and LTR10B inserted and became fixed in intron two of the ancestral Platyrrhini *TRIM22*, where it is fixed in all species sampled.

#### *TRIM5*

In contrast to the three genes of this locus discussed above, Haplorhini *TRIM5* sequences exhibit a significant amount of transposable element associated sequence divergence (Figure 6). Thirty independent *Alu* insertions are found in the *TRIM5* sequences examined. Of these, five are conserved amongst all Haplorhini sequences examined, while 25 are restricted to specific lineages; 11 of these are fixed in multiple species, while 14 are present in a single species. Neither the absolute number of *Alu* insertion events nor the lineage distribution in host genomes is dramatically different than either *TRIM34* or *TRIM22*. A combination of at least 10 large deletion events in addition to numerous insertions cause the near complete replacement of both the first and fourth intronic sequences in Haplorhini primates. Only short regions of homology are conserved in all ten primate species. In the first intron, these regions consist of a 185-nucleotide region immediately following the first exon and a 730-nucleotide region immediately adjacent to the second exon, which includes sequence of LINE L2 origin. In the fourth intron, the conserved regions consist of 310 nucleotides immediately adjacent to the fourth intron, less than 50 nucleotides of LINE L1-derived sequence in the internal region of the intron, and approximately 2200 nucleotides at the 3′ end of the intron. Interestingly, the majority of the transposable element content gained and lost from the first and fourth introns of *TRIM5* is derived from a single class of transposable elements in both cases, these being ERV elements in the first intron and LINE L1 elements in the fourth intron.

Represented in the *TRIM5* genes examined here are a total of ten ERV insertion events containing 11 LTR elements. None of these are conserved in all ten species examined as the result of lineage-specific deletion events excising one or more LTR elements. While all other ERV-derived sequences in all genes examined are solo LTRs (formed as the result of homologous recombination between the 5′ and 3′ LTRs of an ERV element), the HUERS-P3b sequence found in Platyrrhini primates is composed of internal ERV sequences linked to their 5′ LTR9B element that is present in all species except human, chimpanzee and squirrel monkey. HUERS-P3b elements are non-traditional, replication-defective endogenous retroviral-like elements comprised of various fragments from what were at one time replication-competent ERVs. They contain a central LTR8A element, and are flanked by 5′ and 3′ LTR9B elements. The Platyrrhini *TRIM5* HUERS-P3b sequence, in conjunction with its LTR9B 5′ LTR, is found in the negative orientation with respect to *TRIM5* transcription and is missing ∼2500 nucleotides from its 3′ terminus, including the 3′ LTR. In squirrel monkeys, two separate deletion events have removed from this element the entire LTR9B 5′ LTR as well as a significant portion of the internal LTR8A. In contrast, a solo LTR9B element is found in Old World primates consistent with homologous recombination between the 5′ and 3′ LTRs of the original intact HUERS-P3b retroelement and complete loss of the internal sequences.

### The Prosimian *TRIM6/34/5/22* Locus has an Altered Chromosomal Architecture and Contains Less Transposable Element Content than Haplorhini Primates

**Figure 7:**
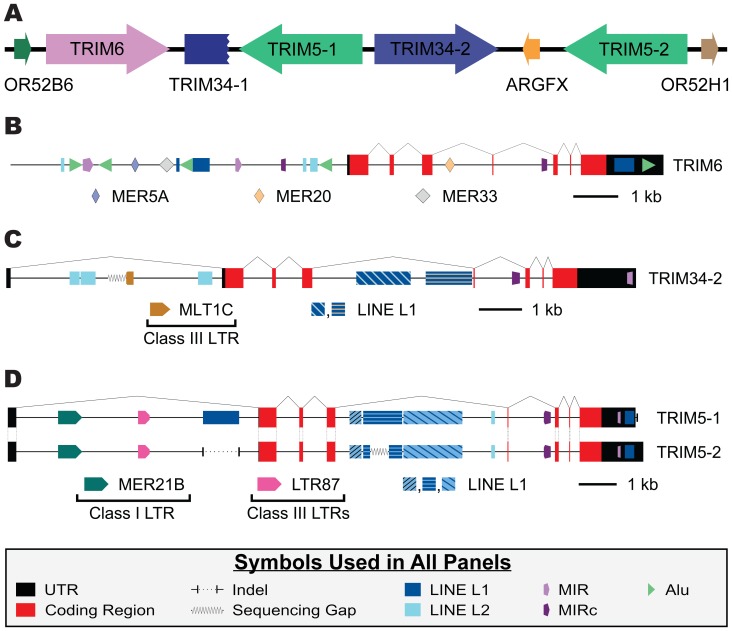
The grey mouse lemur *TRIM6/34/5* genomic locus exhibits a novel architecture, while the genes largely maintain ancestral transposable element content. Panel (A) depicts the relative location and orientation of genes and pseudogenes present in the *TRIM5* genomic locus of the grey mouse lemur. Similar to Figures 5 and 6, RepeatMasker was used to identify repetitive elements present in genes of this locus and graphical representations overlaying the identified transposable elements on the exon/intron structure of *TRIM6* (B), *TRIM34-2* (C), and *TRIM5-1* and *TRIM5-2* (D). Symbols representing non-conserved transposable elements as well as 1 kb scale bars are presented in the panel with which they are associated, while symbols common to all genes are shown at the bottom of the figure.

We sought to determine if the differences in transposable element content and intronic turnover seen in Haplorhini species was a universal characteristic of these genes in primates. Thus, we examined this locus in the grey mouse lemur (*Microcebus murinus*). In this species, we discovered a *TRIM6/34/5* locus with much different architecture than that of Platyrrhini or Catarrhini (Figure 7A). In the grey mouse lemur a combination of a gene duplication event along with a deletion event has resulted in the presence of two *TRIM34* and *TRIM5* genes and the loss of *TRIM22*. Following this duplication, there appears to have been a loss of exons 5 through 8 of the *TRIM34-1* gene, leaving a partially encoded pseudogene. Confirmation of this genomic structure was made through gene specific PCR amplification of intergenic sequences (data not shown). Such a genomic architecture, with multiple *TRIM5* genes, has not previously been reported in primates, although expansions of *TRIM5* copy number are found in mice, rats, and cows [4], [6].

While the novel architecture of the grey mouse lemur’s *TRIM5* locus was unanticipated, the extant genes of this locus can still serve as an outgroup for the previously characterized Haplorhini TRIM genes. In this regard, transposable elements were identified and mapped back onto the mouse lemur TRIM genes (Figures 7B-D). A sequence corresponding to the first exon of *TRIM6* could not be identified in the grey mouse lemur genome sequence, therefore we included in our analysis sequences extending approximately 1500 nucleotides upstream of the promoter proximal MIR element, which is present in all *TRIM6* genes examined (Figures 5A and 7B). In comparison to the Haplorhini sequences, none of the *Alu* elements in the grey mouse lemur genome are found in any Haplorhini sequences (i.e., all 5 *Alu* elements present are unique to the lemur). Additionally, while the lemur sequence contains an MER33 element in the first intron, it lacks the second MER33 element present in the 3′ UTR of Haplorhini species (Figure 5A). With those exceptions, the transposable element content of the mouse lemur *TRIM6* is nearly identical to what is fixed in all Haplorhini sequences examined (Figures 7B, 5A).

Similar to *TRIM6*, all of the transposable elements present in the lemur *TRIM34-2* gene are also found as conserved elements in Haplorhini *TRIM34* sequences (Figure 7C). Notably absent from *TRIM34-2* is the LTR87 insertion fixed in the first intron of all Haplorhini *TRIM34s* examined and also identified in many *TRIM5* sequences. This element appears to have been lost from grey mouse lemur *TRIM34* genes prior to gene duplication as the result of a deletion event, as flanking sequences are also missing (data not shown).

Finally, the *TRIM5* genes from the grey mouse lemur harbor fewer transposable elements compared to their Haplorhini counterparts; of those present, most (9 of 10) are also found in Haplorhini *TRIM5* sequences (Figures 6 and 7D). The only grey mouse lemur specific transposable element is found in the first intron of *TRIM5-1*, a LINE L1 element, which was inserted after gene duplication in this lineage. Of the two ERV LTRs identified in the first intron of the *TRIM5* genes, LTR87 is found in all Haplorhini species studied except human and chimpanzee, while the MER21B element is only found in Cercopithecidae and gibbon. These two LTR elements appear to be the minimal complement of LTRs present in the first intron of *TRIM5* at the time of divergence of Haplorhini from Strepsirrhini. The fourth intron of both mouse lemur *TRIM5s* contain three LINE L1 elements. Fragments of all three can be found in all Haplorhini species. The Platyrrhini LINE L1 content in this intron most closely resembles that of the lemur, while the Cercopithecidae have largely lost these LINE L1 sequences in a series of deletions.

### Gene-specific Differences in Fourth Intron Size and LINE L1 Content

**Figure 8:**
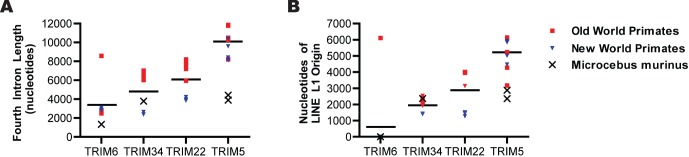
LINE L1 associated evolution in the fourth intron. The number of nucleotides of LINE L1 origin (A), and the size of the fourth intron (B) were calculated for each of the TRIM genes examined. In both panels, the red dots represent the fourth intron of New World primates, while the blue dots represent the fourth intron of Old World primates. The black bars represent average values. Statistical significance was calculated using the Friedman test, a one-way repeated measures ANOVA without assuming Gaussian distributions and using the Dunn’s post-test to compare all genes against one another. A p-value of less than 0.05 is denoted by *, a p-value less than 0.01 is denoted by **, a p-value less than 0.001 is denoted by ***.

This analysis revealed an intriguing phenomenon in the fourth intron of the Haplorhini genes in the *TRIM6/34/5/22* locus. This intron is of interest because in the positively selected antiviral genes the fourth intron spatially separates the exons under positive selection (exons 6–8) from those under purifying selection (exons 2–4) [4], [5], [45]. We observed that in Haplorhini species the length of the fourth intron correlates with the previously published degree of diversifying selective pressure placed on the genes [4]. The average length of the fourth intron in Haplorhini primates is as follows: 3387 nucleotides in *TRIM6*, 4809 nucleotides in *TRIM34*, 6078 nucleotides in *TRIM22*, and 10098 nucleotides in *TRIM5* (Figure 8A). Concomitant with the increase in the length of the fourth intron has been an increase in the amount of LINE L1-derived content within this intron (Figure 8B). Thus, the *TRIM6* from most Haplorhini species lack any LINE L1 sequences, while Haplorhini *TRIM34* averages 1950 nucleotides, *TRIM22* averages 2875 nucleotides, and *TRIM5* averages 5222 nucleotides of LINE L1-derived sequence.

### Over Representation of Newly Inserted LTR Elements in TRIM22 and TRIM5 Compared to TRIM34

One of the most striking differences in terms of transposable element content between the four *TRIM* genes studied is the abundance and variability of ERV elements in *TRIM5*, and to a lesser extent *TRIM22*, compared to the other genes. Nine unique ERV elements were found to have been fixed in *TRIM5* genes during primate evolution. The HUERS-P3b element in Platyrrhini species contains an internal LTR8A, so these nine ERV elements result in ten novel LTR elements. During the same evolutionary time period, eight unique LTR sequences were fixed in *TRIM22* genes, three unique LTRs were fixed into *TRIM34*, and no LTRs were fixed in *TRIM6* (Figures 5 and 6; Table 1). Of the five LTR elements present in primate *TRIM34* (Figure 5B), the LTR87 and MLT1K fragments could be identified in the same relative location in the *TRIM5* loci from other mammalian species, including cats (data not shown). This argues that fixation of these two LTRs predates both the gene duplication event(s) that gave rise to *TRIM5* as well as the speciation events that separated carnivores from primates, thus they were not counted in this analysis. No other LTRs from any of these genes were found to be conserved in both primate and non-primate TRIM gene sequences (data not shown).

**Table 1:** Number and orientation of unique *Alu* and LTR elements captured during primate evolution.

	*Alu*			LTR		
	+	−	Percent Positive	+	−	Percent Positive
*TRIM6*	8	5	61.5	0	0	N/A
*TRIM34*	12	12	50	0	3^a^	0
*TRIM22*	10	17	37	2	6	25
*TRIM5*	16	15	51.6	6	4	60

a LTR elements conserved in both primate and non-primate *TRIM* gene sequences were omitted from quantitation.

Previous studies report a strong bias against LTRs within transcribed genic regions being in the positive orientation relative to the harboring gene, with approximately one in five LTR elements in the positive orientation. *In vitro* experiments suggest that this orientation bias occurs by negative evolutionary selection post-insertion and not as a result of bias during integration [62], [63], [64]. We therefore examined the orientation of primate-specific LTR elements in *TRIM34*, *TRIM22* and *TRIM5* (Table 1). Due to an inversion event resulting in a tandem but oppositely oriented repeat, the MLT1C element present in the fourth intron of *TRIM22* was counted as two elements, one in the positive orientation and one in the negative orientation. It is clear from Table 1 that the orientation of LTR elements in the TRIM genes is non-uniform across genes. None of the three primate-specific LTR insertions into *TRIM34* are in the positive orientation. Two out of eight LTRs in *TRIM22* are in the positive orientation, although one of those is part of the previously mentioned MLT1C element, consistent with the previously reported ∼20% orientation bias for genic LTR elements. In contrast to both *TRIM34* and *TRIM22*, greater than half of the LTR elements present in the *TRIM5* gene are found in the positive orientation. This is similar to the 50% probability of positive orientation based exclusively on integration. No orientation bias was observed for *Alu* elements in genic regions of Haplorhini *TRIM5* (Table 1).

### Differential Transcriptional Regulation of *TRIM22* Mediated by Fixation of an Intronic LTR10D Element

Transcriptional regulation of the *TRIM* genes involved in this study has not been the subject of detailed investigation. Of these genes, the regulation of *TRIM22* transcription has been the best characterized. It has been reported that expression of human *TRIM22* is upregulated upon p53 induction, that a p53-binding site is located in the first intron of this gene, and that mutations abolishing p53 binding to this site result in the loss of p53 responsiveness [65]. Following this, the p53-binding site was found to be located in an LTR10D element, and that a network of p53-regulated genes exists as the result of fixation events involving either the LTR10 or MER61 families of ERV elements [57]. While these elements can be found in both Old and New World primate genomes, an LTR10D is only present in *TRIM22* of Old World primate lineages (Figure 5C). Based on the previous reports and our results demonstrating the presence of this element in Old World primates and absence of this element in New World primates, we hypothesized that *TRIM22* of Old World primates would respond to p53 induction by upregulating transcriptional levels, while *TRIM22* in New World monkeys would be refractory to p53 activation. In order to test this hypothesis, *TRIM22* expression changes were assessed following induction of DNA damage, which is known to induce p53 [57], [65], in peripheral blood mononuclear cells (PBMC) from healthy humans, rhesus macaques, and squirrel monkeys.

**Figure 9:**
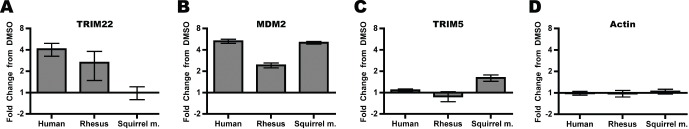
Transcription of *TRIM22* from different primate species is differentially regulated following p53 induction. Following a 3-day stimulation with PHA, PBMCs from humans, rhesus macaques, or squirrel monkeys were treated with doxorubicin or DMSO control for 24 hours. Total RNA was harvested and levels of (A) *TRIM22*, (B) *MDM2*, (C) *TRIM5*, and (D) *β-actin* mRNA were assessed using SYBR green-based qPCR with 50ng per reaction input RNA. Shown are the fold changes measured following drug treatment compared to DMSO controls, as calculated using the ΔΔC(t) method. Error bars represent ± SEM from three independent experiments with *n* = 7 human subjects, *n* = 6 rhesus subjects, and *n* = 4 squirrel monkeys subjects.

Pan-primate quantitative one-step RT-PCR was used to detect changes in *TRIM22* mRNA expression (Figure 9A). In accordance with previous work [57], [65], [66], *TRIM22* expression levels in human and rhesus PBMCs displayed a significant increase in expression of 4.09- and 2.64-fold, respectively. In contrast, no significant change in *TRIM22* RNA levels was observed in squirrel monkey PBMCs, although treatment with 5-fluorouracil tended to reduce *TRIM22* expression. Thus, the general pattern of *TRIM22* expression level changes following p53 induction was consistent with the presence or absence of the p53-binding site containing LTR10D element in a given species.

In order to verify that the changes to *TRIM22* expression were specific for this gene, RNA levels were measured for the control genes murine double minute 2 (*MDM2*), *TRIM5*, and *β-actin*. MDM2 is a well-studied inhibitor of p53 activity whose expression is directly controlled by p53 activation [67], [68], [69], while transcription of *TRIM5* and *β-actin* have been shown to be largely unaffected by DNA damage [66]. Thus, *MDM2* expression serves as a positive control for p53 induction, while *TRIM5* and *β-actin* serve as indicators of global transcriptional changes. When *MDM2* expression levels were assessed, it was observed that this gene was upregulated in the PBMCs of all three species (Figure 9B), demonstrating that the differential transcriptional regulation seen for *TRIM22* was not due to a failure to induce p53 using the DNA-damaging agents. In contrast, *TRIM5* and *β-actin* expression levels remained largely unchanged in PBMCs following DNA damage (Figure 9C-D). These results clearly demonstrate that *TRIM22* is specifically upregulated in human and rhesus PBMCs following p53 induction, and that the p53-binding site in the first intron does not mediate transcriptional regulation of neighboring genes.

## Discussion

Pathogenic invaders can impose major selective pressures on genes involved in the antimicrobial response. Variability in *TRIM5,* and the genomic locus in which it resides, reflects the consequences of such selective pressures. This is embodied by: the variation in copy number of *TRIM5*, *TRIM22*, and *TRIM34* between species [5], [6], [26], elevated rates of non-synonymous change in the coding sequence of *TRIM5* and *TRIM22* [4], [5], [45], maintenance of balanced polymorphisms as well as cross-species sharing of polymorphisms in *TRIM5* [70], [71], and convergent evolution of exon replacement via cDNA retrotransposition [36], [72], [73], [74], [75], [76], [77].

For the four *TRIM* genes studied here, we found that the degree of nucleotide divergence between species for each gene parallels the dN/dS rate ratio previously reported for the coding regions of that gene [4], [61]. For example, *TRIM6*, the gene in this locus with the lowest dN/dS rate ratio, was observed to have on average 91% nucleotide identity between Haplorhini species. In contrast, *TRIM5* has been shown to have the highest dN/dS rate ratio, and genomic sequences had dramatically lower conservation (with only 65% average nucleotide identity between the species included in this study). This high level of diversity correlated with elevated rates of transposable element turnover, much of which was due to alterations in either ERV LTR content in the first intron or LINE L1 content in the fourth intron. In between these extremes, *TRIM22* and *TRIM34* were both found to have between 83% and 85% nucleotide identity in these species. Both of these genes feature a large deletion removing much of the LINE L1 content in the fourth intron. However, in contrast to *TRIM34*, *TRIM22* contains lineage-specific LTR insertions in the first intron.

It is possible that positive selective forces on the coding regions of *TRIM5* and *TRIM22* have indirectly lead to the differences in fixation of transposable elements observed. This is possibly the case for the evolutionary forces acting on the fourth intron of *TRIM22* and *TRIM5*, where increasing LINE L1 content and associated intronic length (3387 nucleotides in *TRIM6*, 4809 nucleotides in *TRIM34*, 6078 nucleotides in *TRIM22*, and 10098 nucleotides in *TRIM5*) are correlated with increasing positive selection of the B30.2 coding region. Taken together, we hypothesize that the fourth intron may function as a “flexible spacer” between the evolutionarily conserved exons encoding the RING, B-box, and coiled-coil domains and the rapidly evolving B30.2 domain.

Differential fixation of LTR elements in the first intron of the *TRIM5* and *TRIM22* genes is unlikely to be solely driven by positive selection in the coding domains. Three main arguments can be made against this. First, the entire coding regions of these genes have not been under uniform selective forces during primate evolution. Rather, only the B30.2 domains of these genes have been under strong positive selection [5], [45], and in the case of *TRIM22* this was restricted to Catarrhini species. Moreover, the more than 8 kb that separates the first intron of *TRIM5* and *TRIM22* genes from the B30.2-domain encodes the tripartite motif, which has been under purifying selective pressure during primate evolution. Second, the differential fixation of LTR elements is restricted to *TRIM22* and *TRIM5* from Haplorhini species and the majority of the LTRs fixed in these species predominantly reside in the promoter-proximal first intron. If the fixation of these elements was driven purely through linkage with positively selected polymorphisms in the B30.2 domain encoding exons one would expect them to be more evenly distributed throughout the genes. Third, the capture of ERV elements is unique to primates; other mammalian species whose *TRIM5* orthologs have been under positive selection have not diversified in this way (data not shown). Thus we believe it is unlikely that the insertion of LTR elements was solely the result of linkage disequilibrium with the positively selected B30.2 domain.

Intriguing correlative evidence points to the evolutionary forces that have resulted in the fixation of LTR elements as being unique to Haplorhini. In these species, genes of the *TRIM6*/*34*/*5*/*22* locus lie adjacent to one another in the following order: *TRIM6*, *TRIM34*, *TRIM5*, *TRIM22*. Three of these genes, *TRIM6*, *TRIM34*, and *TRIM22*, are oriented such that transcription proceeds in the same direction, while transcription of *TRIM5* occurs in the opposite direction (Figure 1A). As a result, in Haplorhini primates *TRIM5* and *TRIM22* are situated in such a manner that only ∼5 kb separates the transcriptional start sites of these genes. While extensive promoter mapping studies for these genes have not been conducted, whole genome mapping has localized H3K27Ac, DNase hypersensitivity, and transcription factors to the sequences surrounding the first exons of both *TRIM5* and *TRIM22*, localizing the core promoter domains to these regions [78], [79]. In Haplorhini primates, both *TRIM5* and *TRIM22* possess intracellular antiviral functions and over the course of primate evolution likely faced multiple rounds of challenge from viruses with divergent tissue tropisms, that required flexibility in matching TRIM gene expression to the target tissue of the challenge virus. The relatively short intergenic region providing core promoter function for both of these genes would serve to limit the evolutionary flexibility of the core promoter, but fixation of LTR elements proximally to this region in *TRIM5* and *TRIM22* could have served as a novel adaptive evolutionary mechanism to modulate transcriptional requirements placed upon these apposed genes.

The capture of LTR sequences into the TRIM locus is not a significant feature of non-primate mammalian species [Diehl, W.E. unpublished data] and is even very limited in prosimians. However, in these non-primate species, there is no evidence for TRIM22 possessing antiviral properties. Indeed, independent deletion events in multiple non-primate species, including all those known to have undergone *TRIM5* gene duplication (mouse, rat, cow, and grey mouse lemur), have resulted in the loss of *TRIM22* [4], [6], [26]. Similarly, recent analyses of the porcine genome show that it encodes only *TRIM6*, *TRIM34*, and a single copy of *TRIM5* [Diehl, W.E. unpublished data]. Thus a functional role favoring maintenance and adaptive evolution of *TRIM22* may be restricted to Haplorhini species. Combined, these observations suggest that a gain of antiviral function for *TRIM22* in primates in combination with the restricted flexibility of the genomic organization of this locus favored serial fixation of ERV LTRs in the promoter proximal regions of these neighboring genes.

There is a growing body of literature demonstrating the profound influences, both at the cellular and organismal level, mediated by alterations in non-coding DNA content, in general, and ERV LTRs, in particular [80], [81], [82], [83], [84], [85], [86], [87], [88], [89], [90]. In humans, well known examples of such regulation include: the human salivary amylase gene whose expression in the salivary glands is mediated by an HERV-E 5′ LTR, which acts as an enhancer element for a cellular promoter [88] and the human fetal gamma globin gene where an ERV9 LTR element ∼40 kb upstream acts to stimulate expression during fetal development and suppress expression after birth [87]. A recent report has shown that a similar ERV LTR capture event has occurred in an antiviral gene in certain lineages of mice, including C57BL. In these strains, a xenotropic murine leukemia virus insertion into the second intron of apolipoprotein B mRNA-editing, enzyme-catalytic, polypeptide-like 3 (*APOBEC3*), resulting in a greater than 4-fold increase in expression in the spleens of mice, and in conjunction with other changes, resulted in an enhanced antiviral phenotype in these mice [58]. Such findings clearly demonstrate that ERV elements and other non-coding DNA, frequently described as “junk DNA”, may indeed contribute to evolution of biological function(s). The recent report of trans-species balancing selection on the LTR12D and PABL_A elements in the first intron of *TRIM5* in both humans and chimpanzees [70] indicating an evolutionary benefit from both the ERV elements as well as the conserved polymorphisms within these elements. This observation thus provides additional evidence for a positive role of LTRs in modulating function within the TRIM locus.

In exploring the cross-species differences in transcriptional control the ERV elements provide to the neighboring genes we focused on *TRIM22*. This gene provides a more tractable model due to its limited transposable element diversity as well as the existence of studies examining its transcriptional regulation. These previous studies demonstrated that a p53-binding site, found in the LTR10D element located in the first intron, is necessary and sufficient for providing p53 responsiveness to this gene in human cells [57], [65], [66]. Because this LTR10D element is present in Catarrhini *TRIM22* but is absent from that of Platyrrhini, we tested the hypothesis that the presence or absence of this element might alter p53 responsiveness at a species level. Indeed, we found that p53-mediated transcriptional control of *TRIM22* expression in response to DNA damage in PBMCs was restricted to those species harboring the LTR10D element. Thus, it does not appear that New World monkeys have acquired p53 responsiveness for *TRIM22* via some other mechanism. In contrast *TRIM5* expression was unaffected by p53 upregulation in all species tested, and while these *TRIM5* genes contain multiple different ERV insertions, all lack an LTR10D element. Together, these findings clearly demonstrate a lineage-specific, differential regulation of transcription resulting from fixation of an ERV LTR element fixed within an antiviral *TRIM* gene. By extension, we would argue that all of the LTR elements fixed in *TRIM5* and *TRIM22* have the potential to alter transcriptional regulation of these genes in response to specific stimuli, e.g. DNA damage, or in a tissue- or lineage-specific manner as demonstrated for murine *APOBEC3* [58]. The regulatory control exerted by intronic LTR elements present in *TRIM5* and *TRIM22* provide the opportunity for a complex interaction between the core promoter and various LTR elements present in a specific gene. The potential for their involvement in transcriptional regulation represents a previously unrecognized consequence of the evolutionary pressures placed on these *TRIM* genes. A better understanding of the genetic basis for transcriptional control of these genes will assist in defining how TRIM5 and TRIM22 exert their antiviral effects *in vivo*.

## Methods

### Samples from Human and Non-human Primates

Human blood samples were obtained from volunteers enrolled in the Emory University Institutional Review Board approved “Emory Vaccine Center’s Healthy Adults Study” (Protocol #555–2000). Volunteers in this study were healthy adults, aged from 23 to 62, who had signed a written informed consent form.

Rhesus macaque and squirrel monkey blood samples were obtained in accordance with NIH guidelines from animals housed at the Yerkes National Primate Research Center. These animals were maintained in accordance with standards of the Public Health Service Policy on Humane Care and Use of Laboratory Animals and the Emory University Institutional Animal Care and Use Committee. Emory University’s Animal Welfare Assurance Number is A3180-01. In addition the Yerkes National Primate Research Center is fully accredited by the International Association for Assessment and Accreditation of Laboratory Animal Care. All Yerkes nonhuman primates are fed a primate specific diet (Purina) supplemented daily with fresh fruit. The animals are monitored daily for clinical well-being and psychosocial enrichment. The collection of blood for this study was reviewed and approved by the Emory University Institutional Animal Care and Use Committee (protocol #028-2009Y) and the collection procedures conformed to the principles described in the Guide for the Care and Use of Laboratory Animals. All macaques and squirrel monkeys included in this study were unrelated adult males with no obvious chronic illnesses. All of the rhesus macaques included in the study were of Indian origin and SIV negative.

Grey mouse lemur liver necropsy samples were harvested at the Duke Lemur Center in accordance with NIH guidelines from animals who died of natural causes. These samples were provided to us via an MTA agreement with the Duke Lemur Center.

### Gene Identification

Genes of the *TRIM5/6/22/34* cluster from human, chimpanzee, and rhesus macaque were retrieved from the respective genomic sequence databases at the National Center for Biotechnology Information (NCBI). Genome assemblies used for these analyses are as follows: *Homo sapiens*, NCBI Build 36.3 (March 2009); *Pan troglodytes*, NCBI Build 2.1 (December 2003); *Macaca mulatta*, NCBI Build 1.1 (February 2006).

Bacterial artificial chromosome (BAC)-derived sequences of the *TRIM5* locus were also obtained from white-cheeked gibbon (*Nomascus leucogenys*), olive baboon (*Papio anubis*), guereza colobus (*Colobus guereza*), Peruvian red-necked owl monkey (*Aotus nancymaae*), common marmoset (*Callithrix jacchus*), Bolivian squirrel monkey (*Saimiri boliviensis boliviensis*), dusky titi (*Callicebus moloch*), and grey mouse lemur (*Microcebus murinus*). The TRIM gene sequences from these primate species were identified by using NCBI’s trace Basic Local Alignment Search Tool (BLAST), with human TRIM genes as query sequences while searching all available primate sequencing databases. In all cases, sequence data obtained in this manner was generated by the NIH Intramural Sequencing Center’s (www.nisc.nih.gov) Comparative Vertebrate Sequencing Initiative. When available, sequence information from multiple overlapping BAC clones were compiled in order to provide the most complete coverage for all four TRIM genes. Genbank accession numbers for the BAC clones used in this study are as follows: AC198823, AC193710, and AC191889 (gibbon); AC147862 and AC148062 (baboon); AC174629 (colobus); AC183999 and AC174391 (owl); AC148555 and AC148636 (marmoset); AC192681 (squirrel); AC173941 and AC172720 (titi); AC197314, AC172705, and AC183331 (grey mouse lemur).

The genomic sequences used in this analysis include a previously described Owl monkey *TRIM5-cyclophilin A* (*TRIM-Cyp*) fusion gene that arose due to the retrotransposition of a processed *cyclophilin A* mRNA into the seventh intron of *TRIM5* [36], [75]. A similar, independent *cyclophilin A* retrotransposition event immediately 3′ of *TRIM5* in macaques has resulted in a *TRIM-cyp* allele in rhesus macaques [72], [73], [74], [76], [77]. However, as this allele is not represented in the macaque genome sequence, it was not included in the analysis.

### Sequence Analysis

Transcribed sequences in the *TRIM6/34/5/22* cluster corresponding to the human alpha splice variant of *TRIM5*, isoform 2 of *TRIM6*, isoform 4 of *TRIM34*, and isoform 1 of *TRIM22* were compiled for further examination. With the exception of *TRIM6*, these sequences correspond to the longest of the described transcripts. The Genbank mRNA accession numbers corresponding to these transcripts are NM_033034.1, NM_058166.3, NM_021616.4, and NM_006074.3 respectively. Multiple sequence alignments of these sequences were generated by hand alignment in MacClade 4.06. Repetitive elements were identified for each sequence using RepeatMasker Open Source version 3.2.6 [A.F.A. Smit, R. Hubley & P. Green, unpublished data]. RepeatMasker options used were ‘slow’ speed/sensitivity and selecting ‘other mammal’ as DNA source, except in the case of human sequences where ‘human’ was selected as the DNA source. Grey mouse lemur sequences were further analyzed using the CENSOR algorithm [91] to identify lemur-specific repetitive elements.

### Quantifying the Rate of Indel Turnover

In order to quantify indel differences, the multiple sequence alignments for each TRIM gene were broken up into 45 pairwise alignments and extraneous gaps were removed using the Gapstreeze program (www.hiv.lanl.gov/content/sequence/GAPSTREEZE/gap.html). Unsequenced regions in the source sequence were dealt in the most conservative manner possible: by assuming that the missing region exactly matched that of the second species of the comparison.

### Calculating Genetic Diversity

In order to assess the amount and type of genetic diversity observed in the various TRIM genes, we utilized the following calculations. The percent nucleotide identity was calculated using the following formula:
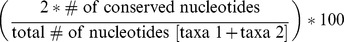



The nucleotide substitution rate was calculated using the following formula:
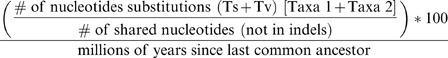
with ‘percentage of sequence per million years’ being the unit of measurement. The rate of indel change was calculated using the following formula:



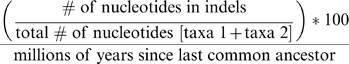
where ‘percentage of sequence per million years’ is the unit of measurement. The rate of indel creation was calculated by dividing the number of indels present in a comparison of two sequences and dividing by the estimated number of years (in millions) since the last common ancestor. In all cases, the dates used for estimation of last common ancestor are the revised dates published in the addendum to Bininda-Emonds et al., 2007 [92].

### Isolation and Culture of Blood Cells

Blood samples were collected in sodium heparin Vacutainer tubes (BD Biosciences; San Jose, CA). PBMCs were isolated following centrifugation of whole blood over a Ficoll cushion. For all experiments, freshly isolated PBMCs were plated at approximately 2×10^6^ cells per well in 6-well plates and cultured for 3 days in RPMI medium containing 15% fetal bovine serum, 10 U/ml penicillin, 10 µg/ml streptomycin, and 3 µg/ml phytohemagglutinin (PHA). Following this stimulation, induction of p53 was accomplished by addition of 0.6 µg/ml doxorubicin to the culture medium. Culture of PBMCs in the presence of 0.1% DMSO served as a carrier control.

### Transcriptional Profiling

At various times following induction of p53, cells were harvested and RNA was isolated using TRIzol (Invitrogen; Carlsbad, CA) according to the manufacturer’s instructions. A one-step, SYBR green-based quantitative RT-PCR was then performed on 50 ng of input RNA. Primer sets targeting *TRIM22*, *TRIM5*, *MDM2*, and *β-actin* were designed and validated to be gene specific, while also targeting regions conserved between all primate species included in this study. The sequences of the primers are as follows: ‘TRIM22 qPCR F’ (5′-ACTGTCTCAGGAACACCAAGGTCA-3′), ‘TRIM22 qPCR R’ (5′-CCAGGTTATCCAGCACATTCACCTCA-3′), ‘TRIM5 qPCR F’ (5′-TGAGGCAGAAGCAGCAGG-3′), ‘TRIM5 qPCR R’ (5′-AGTCCAGGATGTCTCTCAGTTGC-3′), ‘rhTRIM5 qPCR R’ (5′-AGTCCAGGATCTCTCTCAGTTGC-3′), ‘MDM2 qPCR F’ (5′-TGAACGACAAAGAAAACGCCA-3'), ‘MDM2 qPCR R’ (5′-CCTGATCCAACCAATCACCTG-3′), ‘β-actin qPCR F’ (5′-TCGACAACGGCTCCG-3′), ‘β-actin qPCR R’ (5′-TTCTGACCCATGCCCA-3′).

All qPCR reactions were performed with 125 nM forward primer and 250 nM reverse primer concentrations with the following conditions: 55°C for 10 minutes (RT step) and 95°C for 5 minutes followed by 45 cycles of 95°C for 30 seconds, gene-specific annealing temperature for 30 seconds, 72°C for 30 seconds followed by melting curve analysis. The annealing temperatures were as follows: 64°C for *TRIM22*, 56.5°C for *TRIM5*, 61°C for *MDM2*, 57°C for *β-actin*. Relative fold change in gene transcription was calculated via the ΔΔC(t) method using DMSO treatment values as the baseline.

### Genotyping

Human and rhesus macaque subjects had their p53 coding sequence and *TRIM22* LTR10D genotyped. The full p53 coding sequence was PCR amplified from unstimulated PBMC RNA following cDNA generation using an oligo d(T) primer. PCR amplification of p53 was performed using the following primers at 200 nM concentrations: ‘p53 F’ (5′-GGCTGGGAGCGTGCTTTC-3′) and ‘p53 R’ (5′-CACAACAAAACACCAGTGCAGGC-3′).

Reactions were carried out with the high-fidelity Phusion enzyme (New England Biolabs, Ipswich, MA) with the following thermocycler conditions: 95°C for 2 minutes followed by 35 cycles of 95°C for 15 seconds, 62.5°C for 15 seconds, and 72°C for 1 minute 30 seconds and a final 7-minute extension at 72°C. Direct sequencing of this p53 PCR product was performed using the original PCR primers as well as the following primers: ‘p53 480–500 seq F’ (5′-CCTCAACAAGATGTTTTGCCA-3′), ‘p53 576–598 seq R’ (5′-TGTGCTGTGACTGCTTGTAGATG-3′), ‘p53 1021–1039 seq F’ (5′-AACAACACCAGCTCCTCTC-3′), and ‘p53 1081–1099 seq R’ (5′-CACGCCCACGGATCTGAAG-3′).

For the genotyping of the intronic LTR10D, unstimulated human and rhesus PBMCs were used as a source of genomic DNA, which was isolated using the DNeasy Blood and Tissue Kit (Qiagen, Valencia, CA) according to the manufacturer’s protocols. This was used as source material for PCR amplification of the LTR element using ‘TRIM22 LTR10D F’ (5′-GACCATTCATTTCTTCAATCTAGGTAC-3′) and ‘TRIM22 LTR10D R’ primer (5′-ATCAAAATGACAGAATAGGAATGTGGG-3′). PCR was performed with the Phusion enzyme with the following thermocycler conditions: 95°C for 2 minutes followed by 35 cycles of 95°C for 15 seconds, 56°C for 15 seconds, and 72°C for 1 minute 30 seconds and a final 7-minute extension at 72°C. Direct sequencing of the PCR product was performed using the PCR primers.

Rhesus macaques also had their *TRIM5* coding sequences genotyped using previously described methods [74], [77].

Grey mouse lemur DNA was extracted from frozen liver samples using the Wizard Genomic DNA Purification Kit (Promega, Madison, WI), according to the manufacturer’s protocol. This was used as source material for PCR verification of the *TRIM6/34/5* genomic architecture observed in BAC clone sequences found in GenBank. PCR primers were designed to specifically bind to the extreme 5′ and 3′ regions of each gene present in the BAC clones, such that PCR amplifies the intergenic region separating the genes. Presence of PCR product as well as definitive sizing via agarose gel electrophoresis were used as positive indicators of genomic architecture found in GenBank BAC clones. More detailed primer, strategy, and protocol information for these PCR reactions is available upon request.

### Sequence Information

Four unique rhesus macaque LTR10D alleles were discovered (Figure S3 and Table S1) and have been deposited to GenBank with the following accession numbers: allele 1, HM104186; allele 2, HM104187; allele 3, HM104188; allele 4, HM104189. These sequences differ from one another at several nucleotide positions. However, none of these polymorphic sites are located within, or immediately adjacent to, the p53 binding site.

Human and rhesus macaque p53 nucleotide sequences were *in silico* translated and aligned (Figure S4). Amino acid sequences generated in this study match previously described human p53 72P and 72R alleles and the rhesus macaque protein sequence (GenBank accession numbers NP_000537, AAD28628, and NP_001040616, respectively). While only one predicted amino acid sequence was observed, three rhesus macaque p53 alleles were identified. The allele 1 sequence corresponds to that of the rhesus genome sequence (GenBank accession #NM_001047151), and the other two alleles only differ by synonymous polymorphisms. The sequences corresponding to alleles 2 and 3 have been deposited to GenBank with the accession numbers HM104190 and HM104191, respectively.

## Supporting Information

Figure S1 Reduced rate of indel change in TRIM5 from Old World monkeys. Nucleotide sequence alignments were used to calculate the rate of indel change between all pairs of species in *TRIM6* (A), *TRIM34* (B), *TRIM22* (C), and *TRIM5* (D). These analyses have been broken down for comparisons within and between the following evolutionarily distinct groups of primates: hominids, cercopithecidae (Old World monkeys), and platyrrhini (New World monkeys). Dots indicate separate pairwise sequence comparisons and the black bars represent mean values.
(EPS)

Figure S2 No correlation between percent nucleotide identity and the nucleotide substitution rate. The correlation between percent nucleotide identity and the nucleotide substitution rate was examined using linear regression analysis for *TRIM5* (A), *TRIM22* (B), *TRIM34* (C), and *TRIM6* (D). The r^2^ and p-values resulting from the analyses are indicated in each panel.
(EPS)

Figure S3 Amino acid alignment of human and rhesus macaque p53. Amino acid residues highlighted in red correspond to the transactivation domain of p53. Residues highlighted in blue correspond to the DNA-binding domain of p53. Residues highlighted in green correspond to the multimerization domain of p53. The bracketed region indicates the epitope that monoclonal anti-p53 antibody DO-1 recognizes.
(EPS)

Figure S4 Nucleic acid alignment of the LTR10D element found within the first intron of Old World primate TRIM22. Nucleotide sequences comprising and flanking the Old World-specific LTR10D element were trimmed from the greater TRIM22 nucleotide alignment. The sequence corresponding to the LTR10D element is numbered from 1 to 516; sequences upstream and downstream are numbered in reference to this element using – and + symbols, respectively. The red highlighted nucleotides correspond to the target site duplication that occurred during ERV integration. The nucleotides highlighted in green correspond to the p53-binding region. Nucleotides highlighted in blue correspond to the nucleotides found to be polymorphic in rhesus macaques.
(EPS)

Table S1 Allelic nucleotide polymorphisms found in the LTR10D elements within rhesus macaque TRIM22.^ a^Nucleotide positions correspond to those shown in Figure S4.
(DOC)

File S1 Fasta formatted nucleotide alignment of primate genomic *TRIM6* sequences.
(FASTA)

File S2 Fasta formatted nucleotide alignment of primate genomic *TRIM34* sequences.
(FASTA)

File S3 Fasta formatted nucleotide alignment of primate genomic *TRIM22* sequences.
(FASTA)

File S4 Fasta formatted nucleotide alignment of primate genomic *TRIM5* sequences.
(FASTA)
