# Supplementary material for: Elevated Rate of Fixation of Endogenous Retroviral Elements in Haplorhini TRIM5 and TRIM22 Genomic Sequences: Impact on Transcriptional Regulation
Source: PLoS One. 2013 Mar 14;8(3):e58532. doi: 10.1371/journal.pone.0058532 (PMC3597737; doi:10.1371/journal.pone.0058532)
Supplement: Table S1 — Allelic nucleotide polymorphisms found in the LTR10D elements within rhesus macaque TRIM22. aNucleotide positions correspond to those shown in Figure S4. (DOC) [file pone.0058532.s005.doc]

| Genotype | nt –6^a^ | nt 9 | nt 173 | nt 287 | nt 297 | nt 429 | nt 479 |
| --- | --- | --- | --- | --- | --- | --- | --- |
| Allele #1 | C | A | C | G | T | C | G |
| Allele #2 | C | G | C | T | C | C | G |
| Allele #3 | T | A | C | G | T | T | G |
| Allele #4 | C | A | T | G | T | C | T |
